# Scanned Proton Beam Performance and Calibration of the Shanghai Advanced Proton Therapy Facility

**DOI:** 10.1016/j.mex.2019.08.001

**Published:** 2019-08-24

**Authors:** Hang Shu, Chongxian Yin, Haiyang Zhang, Ming Liu, Manzhou Zhang, Liying Zhao, Kecheng Chu, Xiaolei Dai, Michael F. Moyers

**Affiliations:** a Shanghai Institute of Applied Physics, Chinese Academy of Sciences, 2019 Jialuo Road, Shanghai, 201800, China; b Shanghai Advanced Research Institute, Chinese Academy of Sciences, No. 99 Haike Road, Zhangjiang Hi-Tech Park, Pudong, Shanghai, 201210, China; c Shanghai APCTRON Particle Equipment Co. Ltd., 2019 Jialuo Road, Shanghai, 201800, China; d Shanghai Proton and Heavy Ion Center, Pudong, Shanghai, 201315, China

**Keywords:** Scanned Proton Beam Performance and Calibration, Proton therapy, Spot scanning, Technical commissioning, Dosimetry

## Abstract

The Shanghai Advanced Proton Therapy facility (SAPT) is a hospital-based facility that began construction in December of 2014 with commissioning of the first scanned proton beam line starting in October of 2017. Proton beams are extracted from a synchrotron accelerator with energies between 70 and 235 MeV. Beam delivery uses the modulated scanning and energy stacking techniques to produce a maximal scanning area of 40 × 30 cm^2^ at the iso-center. Prior to clinical use, the beam delivery system was characterized and calibrated following the guidelines of the IEC 62667 medical electronic equipment standard including the spot size in air, spot position, depth dose distributions, and lateral dose profiles, as well as the beam monitor calibrations following the IAEA TRS-398 recommendations with small differences.

• The measured dosimetric results showed that the full width at half maximum (FWHM) for the beam spot size in air varied approximately from 6 mm to 13 mm. The dose fall-off (DDF) derived from the measured depth dose in water varied from 4.7 mm at 235 MeV to 0.7 mm at 70 MeV. The homogeneity of the scanned field was better than 2% for various energies as expected.
• Furthermore, the beam reproducibility and proportionality delivery accuracy was also stable with the results better than 0.1% and 1% respectively. Finally, the dose monitor calibration factor, its reproducibility and stability were tested. Reproducibility tests exhibited a standard deviation (SD) result of less than 1% during the test period.
• All the measured dosimetric parameters showed that the design specifications were well achieved and the results are suitable for being used as a part of the clinical commissioning and quality assurance program for treating patients.

## Graphical abstract

**Figure:**
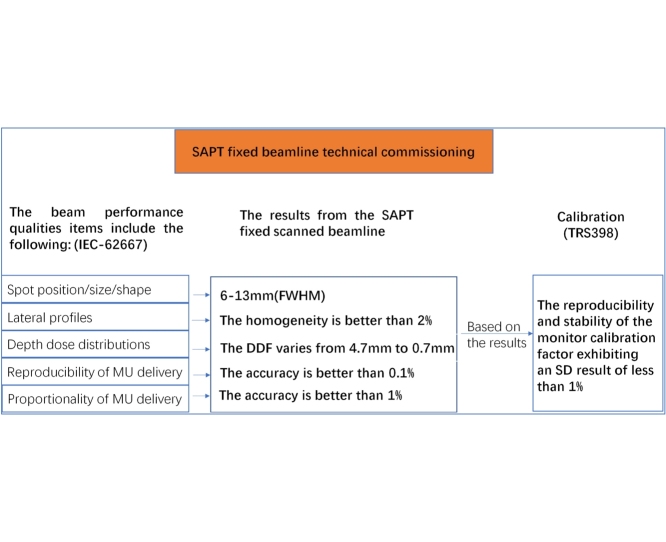

**Specifications Table**

**Table:** 

Subject Area:	Physics and Astronomy
More specific subject area:	Dosimetry
Method name:	Scanned Proton Beam Performance and Calibration
Name and reference of original method:	IEC-62667 medical standard; IAEA TRS-398; J¨akel J¨akel J€ akel O O. Jäkel, G.H. Hartmann, C.P. Karger, P. Heeg, A calibration procedure for beam monitors in a scanned beam of heavy charged particles. Med. Phys. 31, 1009–1013 (2004). DOI: 10.1118/1.1689011

## Introduction

Interest in tumor therapy with protons has been increasing over recent years because the depth of the high-dose Bragg peak of the proton beam is well-defined and little dose is delivered distal to the Bragg Peak. Proton therapy is thus an excellent tool for radiation therapy, especially for those tumors located close to critical organs [[1], [2], [3]]. With this in mind, a hospital-based irradiation facility was designed and built for the treatment of deep-seated tumors using energies up to 235 MeV [4]. This home-made facility comprises five beam lines: one large field fixed beam line, two rotating beam lines (one with 180° and one with 360° rotation capability), one fixed beam line to treat eye tumors with a proton energy of 70 MeV, and one experimental beam line. The first three beam lines use a spot scanning technique while the eye beam line uses a scattering technique. The fixed beam line was the first to be installed with commissioning starting on October 26, 2017. During commissioning, a variety of tools were used to study the beam performance qualities according to the IEC 62667 light ion beam medical equipment performance characteristics standard [5]. This manuscript describes these tools and tests.

## Methods and materials

### Overview of beam delivery system

The beam delivery system uses a modulated scanning technique to spread the protons laterally across a tumor while an energy stacking technique is used to spread the protons across the tumor in depth [6,7]. Using these techniques, protons are accelerated and slowly extracted from the synchrotron directly at the energy required for the tumor without the use of any range shifting material. The user, via the treatment planning system, can select from 94 pre-defined energy levels. During treatment delivery, the energy is changed from the highest energy to the lowest energy necessary to cover the tumor. The protons transported from the accelerator are scanned laterally across the tumor by using two sequential magnets to deflect the spot beam in orthogonal directions. The dose is delivered to the patient as a superposition of spots aimed at discrete locations with the beam being switched off while moving from one location to the next. The beam on/off switch time between the spots corresponds to the radio frequency knockout (RF-knockout) system in the ring of 0.3 ms. The dose delivery is monitored by two parallel-plate ionization chambers (PPIC); one is used as the primary dose monitor to terminate beam delivery at the specified dose while the other is used as secondary redundant dose monitor for safety. A multi-strip ionization chamber measures the beam position and size at each aiming spot.

**Fig. 1:**
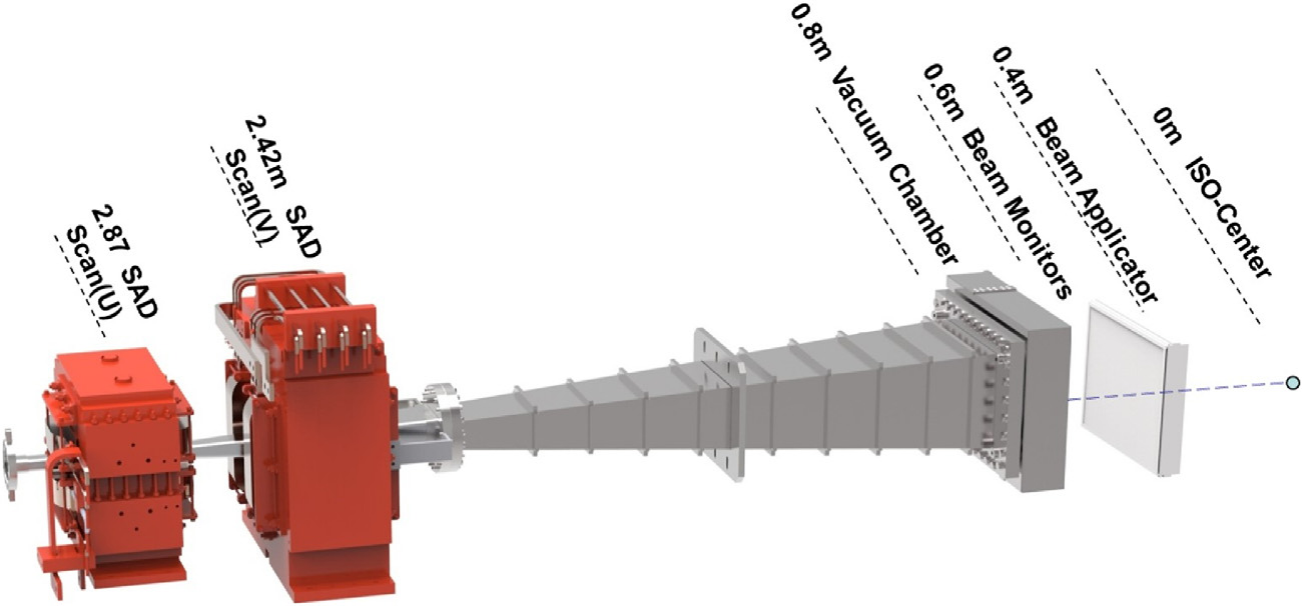
Layout of the SAPT scanning system for fixed beam line.

Fig. 1 shows the layout of the scanning irradiation system for the fixed beam line. The distances from the scanning magnets, Scan (U) and Scan (V), to the iso-center are 2.87 m and 2.42 m respectively. The vacuum window is made of 0.1 mm-thick titanium and located 0.8 m upstream from the iso-center. The primary and secondary dose monitors as well as the position monitors are placed immediately after the vacuum window. Since the lowest beam energy extracted from the accelerator is 70 MeV, the treatment of shallow skin tumors is achieved by inserting a discrete range shifter plate into a beam applicator that can be extended or retracted from the isocenter to maintain a small distance from the patient. When the beam applicator is completely retracted, the distance from the bottom of the beam applicator to the iso-center is 400 mm.

Table 1 shows the main parameters of the beam delivery system. The field size and maximum range of the system were chosen to reduce the typical irradiation times for pediatric spine treatments of children. In order to cover the required large scanning area, the PPIC monitor was designed with a large sensitive area of 367 mm × 276 mm to fit the scanning field of 400 mm × 300 mm. The gap between the electrodes is 5 mm. When a high voltage of −2500 V is applied, the ion charge collection time of the air-filled dose monitor is faster than 70 μs and the collection efficiency is better than 99%. The electronic output of the monitor system is 0.2 pC per count with one monitor unit (MU) being defined as 100,000 counts (2 × 10^−8^ C). The maximum value of MU for any spot is 0.1 MU while the minimum value is 0.005 MU.

**Table 1:** The main parameters of beam delivery system.

Parameters	Value
Energy	70–235 MeV
Energy steps	94 steps
Maximum range	34 g/cm^2^
Field size (U × V)	40 cm × 30 cm
SAD (U, V)	2.87 m, 2.42 m
Scanning speed (U, V)	2 cm/ms, 0.5 cm/ms
Spot size at isocenter in air	6–13 mm (FWHM)
Beam intensity	0.3–3 nA
Dose rate	2 Gy/(L min)

### Scanning spot position, size, and shape

The spot shape is neither circular nor symmetric in the transverse XY plane perpendicular to the beam axis because of the beam optics and the accelerator characteristics. Before the accelerator design was finalized, the fixed scanning delivering system of the horizontal beam line was simulated with the Geant 4.9.3 Monte Carlo code and the result showed that the beam spot size (FWHM) in air at the iso-center would be between 6 and 13 mm, a sufficiently small spot size to produce a large gradient at the edge of the tumor. The performance of the spot position, size and shape with energies of 70 MeV, 130.1 MeV, 161.1 MeV, 179.9 MeV, 202 MeV, 219.2 MeV, and 235 MeV were measured in air at the isocenter plane using both a commercial 2D scintillator (Lynx, IBA Ion Beam Applications S.A.) and a radiochromic film (Gafchromic EBT Film, International Specialty Products, Wayne, NJ).

### Lateral profiles

The homogeneity of the scanned field was checked for various field sizes and energies. For testing the homogeneity of the lateral profile, the Lynx detector was again used. Normally, the required dose homogeneity should be better than 5% in both directions across the treatment field. This degree of homogeneity is also a prerequisite for calibrating the primary dose monitor chamber. In this work, the field used for calibrating the beam monitors is a 60 mm square and calibrated at the multiple energies with 70 MeV, 130.1 MeV, 161.1 MeV, 179.9 MeV, 202 MeV, 219.2 MeV, and 235 MeV. All the testing parameters following the definitions provided in the ICRU 78 report [8].

### Energies, depth doses, and ranges

Important input parameters for treatment plan modelling are the measured depth dose distributions. In this study, these distributions were measured for the preset proton energies with a water phantom (MP30PL, PTW, Freiburg, Germany) and a large diameter parallel plate chamber (Bragg peak chamber Model 34070, PTW). The Bragg peak chamber has a nominal sensitive volume of 10.5 cm^3^ with the water equivalent thickness (WET) of its front window being 0.4 cm. The chamber was specifically designed to measure the depth distributions of single spot proton beams. This large diameter chamber (effective radius of 4.08 cm and an air gap between electrodes of 2 mm) could capture the complete spot including the scattered protons since the spot size of the 70 MeV beam at a depth of 38 mm of water is about 14 mm (FWHM) and the spot size of the 235 MeV beam at a depth of 340 mm is about 18 mm.

### Reproducibility of MU delivery

In order to measure the reproducibility of MU delivery with different dose rates, a single spot was delivered from the radiation head and then passed through the Bragg IC (TW34070, PTW) which was read out with an electrometer (UNIDOS-E, PTW). The beam intensity, i.e. monitor dose rate, was varied from the highest (10 MU/s) to the lowest (1 MU/s) to compare the dose obtained from the dose monitor and the Bragg IC.

### Proportionality of MU delivery

The MU proportionality was also measured with the Bragg Peak chamber placed in a plastic phantom (PTW) at a depth of 15 mm. The test was performed with MU rates ranging from 1 MU/s to 10 MU/s at 235 MeV, 161.1 MeV and 70 MeV.

### Calibration of beam monitoring chamber

One of the most important aspects of commissioning a new beamline is calibration of the primary monitor chamber. Typically the primary monitor is calibrated in terms of absorbed dose to water as it can serve to verify the calculated results of a treatment plan [9]. The absorbed dose to water was determined using the IAEA TRS-398 (an international code of practice Technical Report Series No.398 published by the IAEA in 2000) reference conditions [10]. The primary dose monitor was adjusted to a specific amount of charge collected, corresponding to a single monitor unit, and then calibrated with each individual pseudo-monoenergetic proton beam. This calibration method is slightly different from the passive delivery method that was used for most of the earlier proton and ion therapy facilities [11,12], but similar to some cases of uniform scanning systems, spot-scanning systems, and raster-scanning systems [[13], [14], [15]]. Positioning of the effective point of measurement with the plane-parallel ionization chamber is taken into account.

Calibration of the primary dose monitor was performed in the plateau region of the mono-energetic proton beam instead of the center of a SOBP (Spread-Out Bragg Peak). The determination of D_w_ (Dose to water) was chosen at Z_ref_ (a reference measurement depth) of 1.5 g/cm^2^ instead of 3.0 g/cm^2^ as recommended by the IAEA TRS-398. This is because on the one hand, z_ref_ should be as shallow as possible so as to minimize the steep dose gradient in the plateau region of low-energy proton beams while, on the other hand, z_ref_ should be deep enough so as to avoid the nuclear build-up region of high-energy proton beams. Taking these two criteria into account, it seems that z_ref_ should be energy dependent — as it is, for instance, in the case of high-energy electron beams. In practical experience, however, better results are achieved with z_ref_ set to a constant value of 1.5 g/cm^2^. A drawback of this method is that it is sensitive to setup errors when positioning the reference point of the chamber.

The reference dosimeter used was a Markus Chamber with a ^60^Co calibration factor in terms of absorbed dose to water provided by the National Institute of Metrology (NIM) of China, the primary standard dosimetry laboratory. According to TRS-398 and AAPM TG 51, the NIM laboratory established the absolute measurement of the absorbed dose to water with ^60^Co in 2013. The laboratory also took part in an international collaboration to achieve traceability of the water absorbed dose for radiation therapy [16,17].

## Results and discussion

**Fig. 2:**
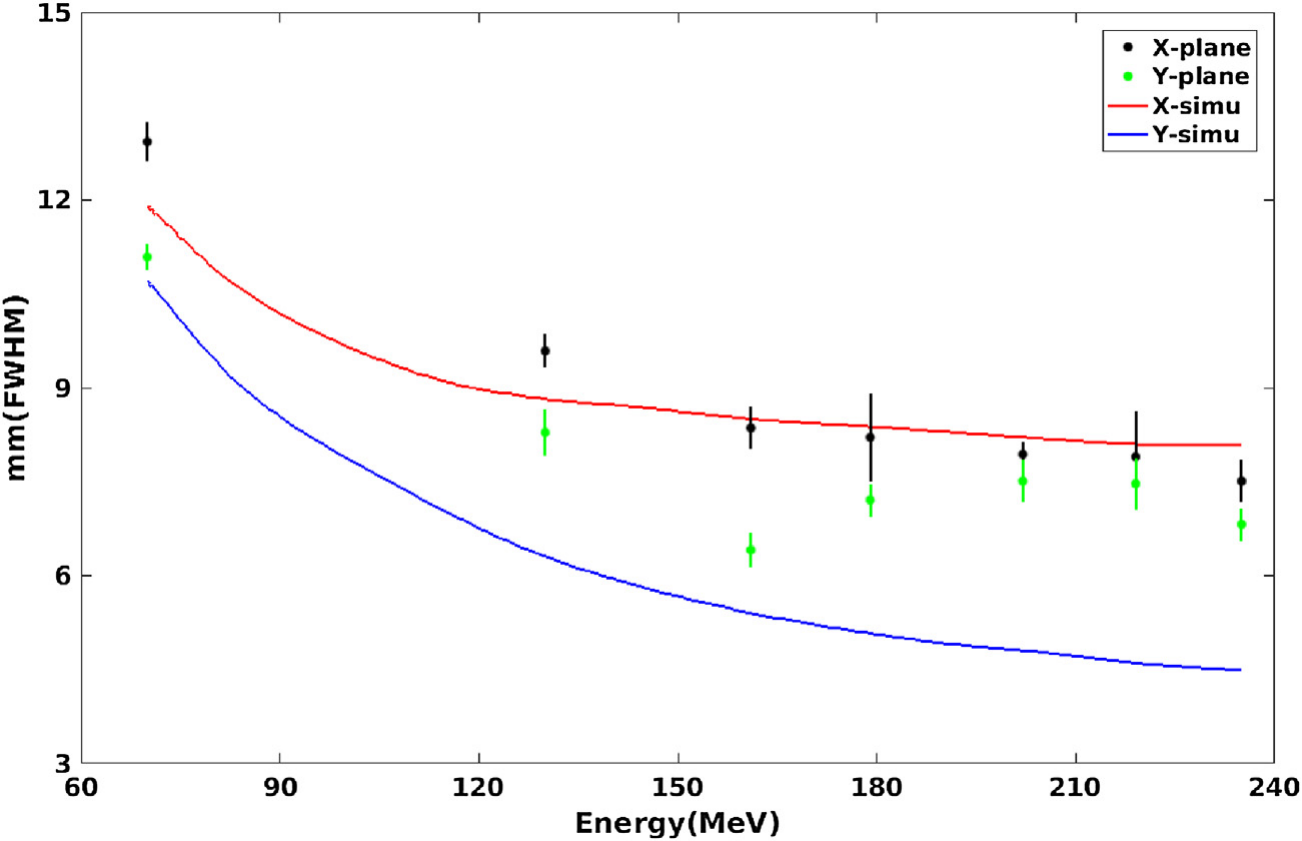
FWHM of lateral profiles in air of single spot beams at the iso-center plane as a function of energy. The solid curves represent the simulation results while the dots represent the measurements.

Fig. 2 shows a comparison of the spot size measurements with the simulation results. The measured spot size at 70 MeV in the X plane was 12.93 mm, nearly 1 mm larger than the largest designed value. In practice, however, the beam at an energy of 70 MeV is only used with the eye beam line and the beam spot for that beam line is additionally scattered to achieve the irradiation field size. Since the large beam spot size is easier to scatter, the beam size was not adjusted to a smaller size at that energy. Although the spot widths in the Y direction could be tuned smaller to achieve a smaller lateral penumbra, to achieve a more uniform dose distribution within the field the spots were tuned to provide a nearly circularly symmetric shape, hence, most of the spot sizes in the Y plane (in Fig. 2) are larger than the designed values and are close to those in the X plane. The measured dosimetric results showed that the full width at half maximum for the beam spot size in air varied approximately from 6 mm to 13 mm.

**Fig. 3:**
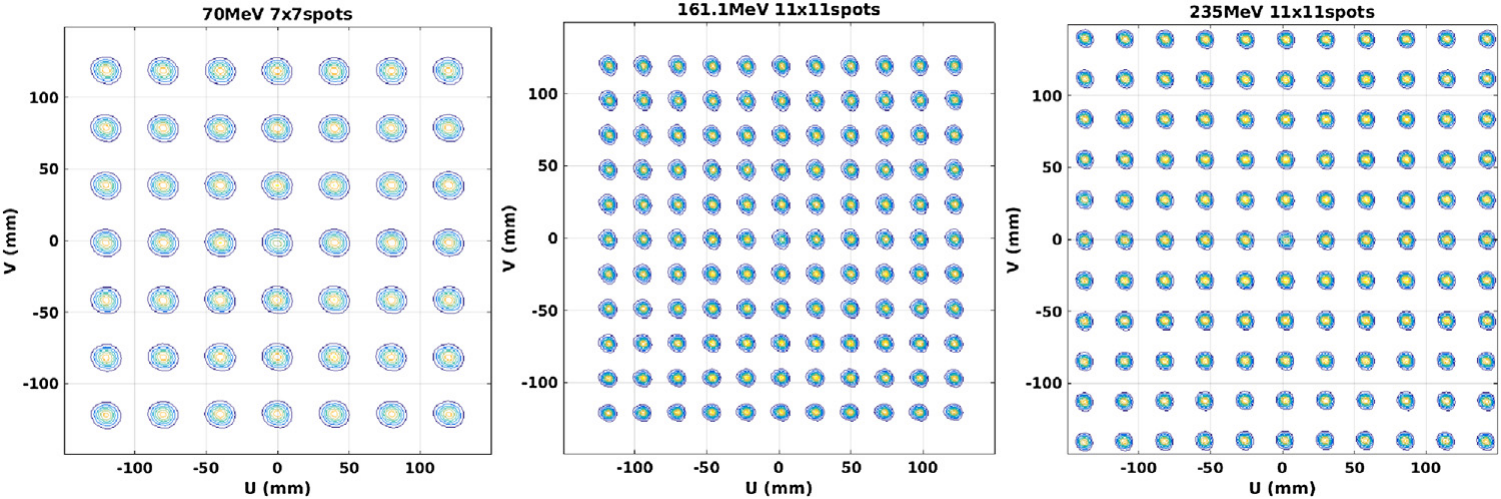
The measurement results of spot matrices at 70 MeV, 161.1 MeV and 235 MeV.

Fig. 3 shows the measurement results of the delivered spot matrices with energies of 70 MeV, 161.1 MeV and 235 MeV. The measured positions of each spot match the planned positions to within 0.5 mm in both the X and Y positions satisfying the specifications. Due to different energies have different spot sizes, the center-to-center spot spacing is varied for different energies to enable accurate measurements of the spot positioning.

**Fig. 4:**
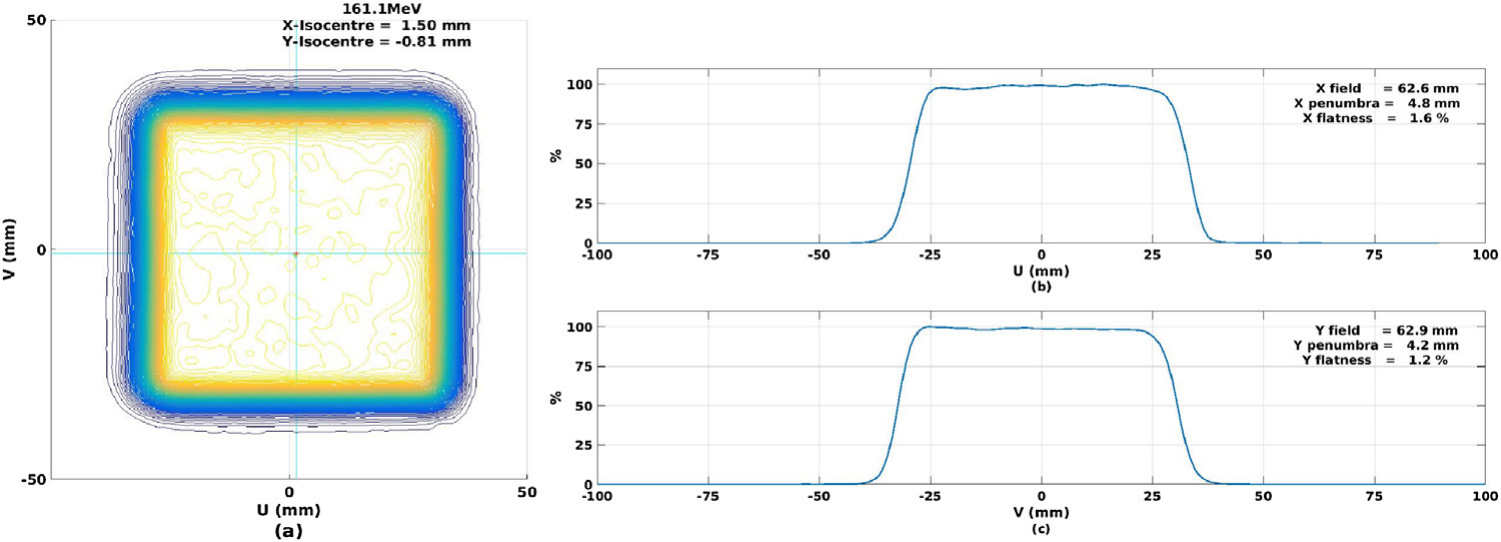
a) shows the homogeneity distributions of the standard calibration field with a 60 mm × 60 mm and the spot spacing with 3 mm × 3 mm at 161.1 MeV. Fig. b) and Fig. c) describe the characteristic of the X and Y planes which illustrate that the homogeneity of the delivered dose in the scanning fields was better than 2%.

Fig. 4 shows an example plot of the homogeneity distributions of the standard calibration field with a 60 mm × 60 mm and the spot spacing with 3 mm × 3 mm at 161.1 MeV. The results illustrate that the homogeneity of the delivered dose in the scanning fields was better than 2%. Table 2 shows the results of the homogeneity tests in the 60 mm × 60 mm calibration field at 70 MeV, 130.1 MeV, 161.1 MeV, 179.9 MeV, 202 MeV, 219.2 MeV, and 235 MeV.

**Table 2:** The homogeneity results of the major axis lateral profiles at 70 MeV, MeV, 161.1 MeV, 179.9 MeV, 202 MeV, 219.2 MeV, and 235 MeV.

Energy (MeV)	X Penumbra (mm)	X Flatness (mm)	Y Penumbra (mm)	Y Flatness (mm)
235.0	5.1	1.4%	4.8	1.4%
219.2	5.2	1.7%	6.5	1.0%
202.0	5.4	1.0%	6.2	0.9%
179.9	5.4	1.3%	5.3	0.9%
161.1	4.8	1.6%	4.2	1.2%
130.1	7.3	1.5%	6.0	0.8%
70.0	9.4	1.3%	8.4	0.5%

**Fig. 5:**
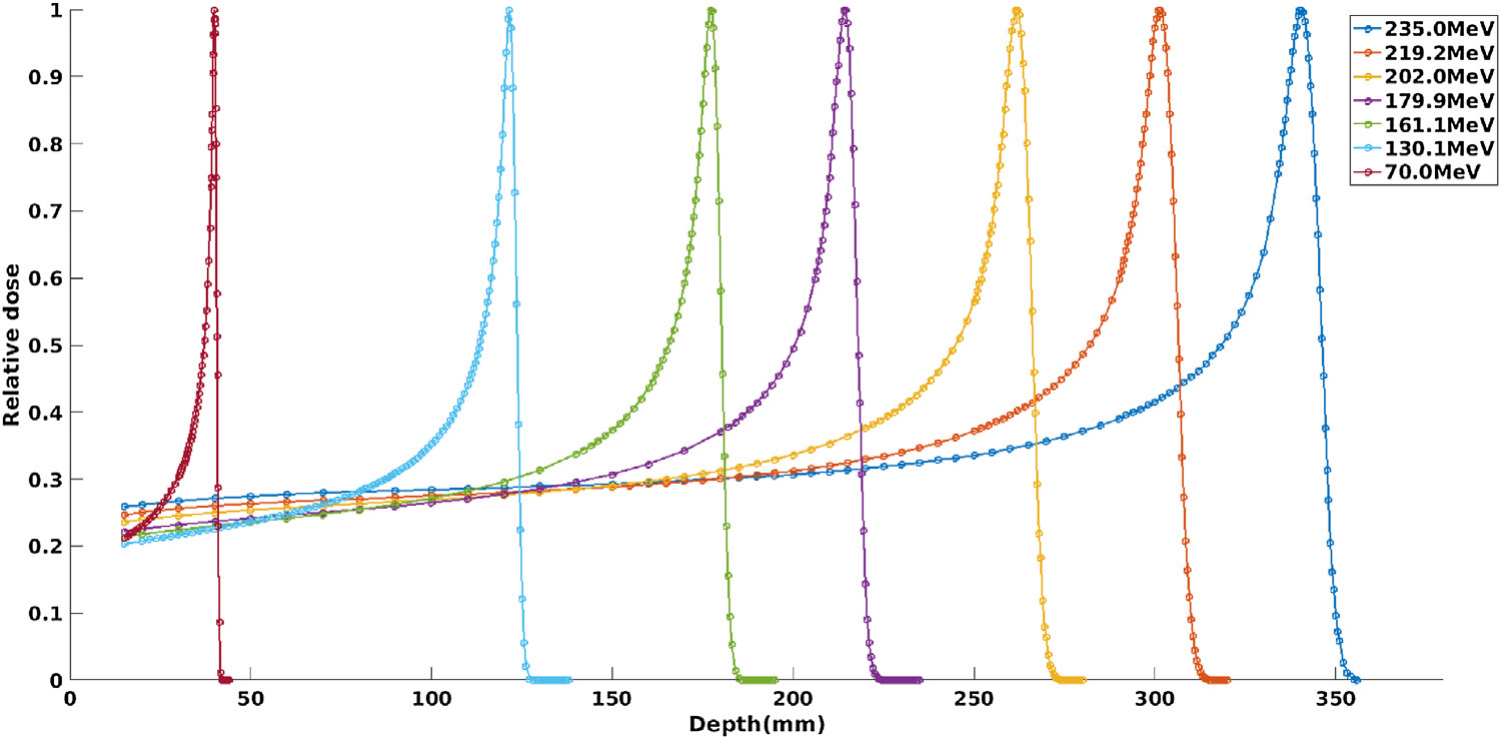
Laterally integrated depth dose distributions measured in water with the Peakfinder at 70 MeV, 130.1 MeV, 161.1 MeV, 179.9 MeV, 202 MeV, 219.2 MeV, and 235 MeV.

Fig. 5 presents the depth dose distributions, illustrating that the width of the peak in the depth direction increases with increasing proton energy. The depth dose distributions are important input parameters for the treatment plan modeling. Parameters associated with the depth dose distributions at each energy are given in Table 3. The definition of the range in the table is the depth along the beam central axis in water to the distal 90 percent point of the maximum dose value (D90), the same definition given in the ICRU 78 report. The DDF 80% - 20% data shows the distal dose fall off at the highest extracted energy was less than 5 mm, mainly because of the narrow energy spread of the extracted spills (less than ±0.1%) in this synchrotron. The narrow DDF could be a benefit in sparing distal healthy organs.

**Table 3:** Parameters derived from the measured depth dose distributions.

Energy (MeV)	Range (g/cm^2^)	Entrance-to-peak dose ratio	DDF (mm)
235.0	34.09	4.7	4.7
219.2	30.17	4.9	4.2
202.0	26.19	5.1	3.8
179.9	21.39	5.4	3.2
161.1	17.67	5.6	2.6
130.1	12.05	5.9	1.9
70.0	3.84	6.4	0.7

**Fig. 6:**
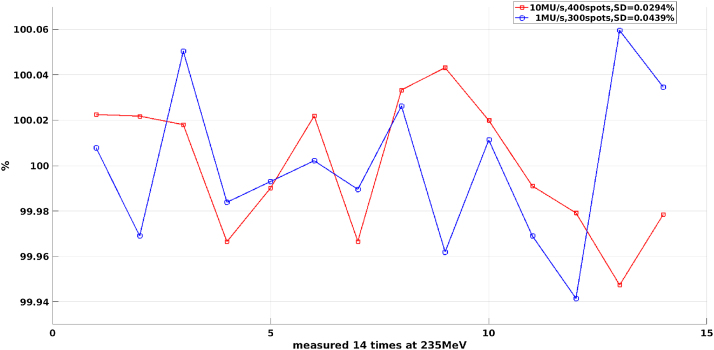
The beam reproducibility delivery accuracy is better than 0.1% at 235 MeV.

Fig. 6 shows the results of reproducibility of MU delivery at an energy of 235 MeV with the highest dose rate (10 MU/s) and the lowest dose rate (1 MU/s). The highest dose rate with 10 MU/s is plotted as a red line, the reproducibility test was repeated 14 times, and each time the delivery consisted of 400 spots with 2500 counts being delivered at each spot. The blue line represents results with the lowest dose rate of 1 MU/s, which was also repeated 14 times and each time delivering 300 spots and 1000 counts at each spot. The results show that the beam delivery reproducibility is better than 0.1% at both the highest and lowest rates.

**Fig. 7:**
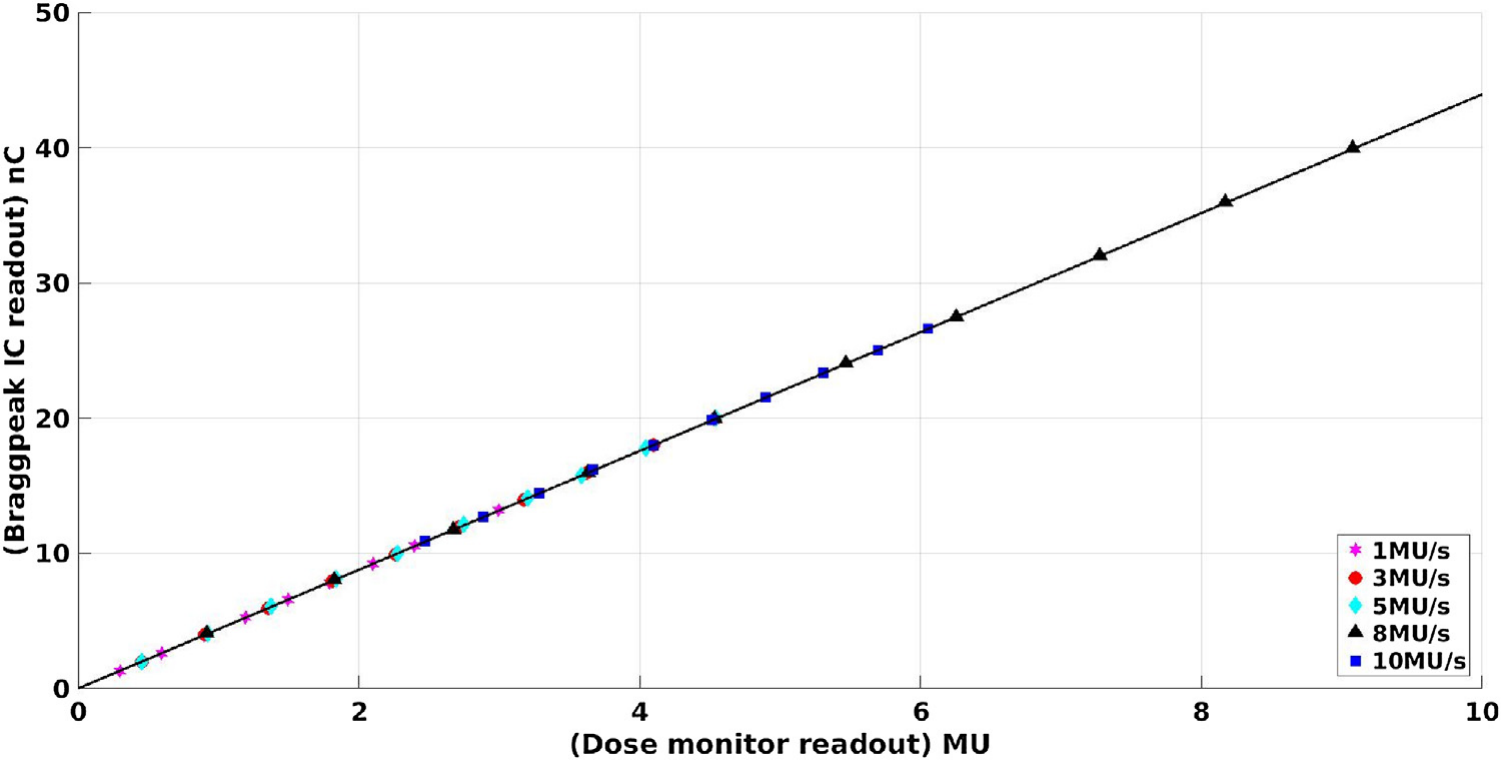
Linear fitting with multiple dose rates of the primary dose monitor.

Fig. 7 shows the MU proportionality test results at 235 MeV with different dose (1 MU to 10 MU with each step of 1 MU) at the different dose rates (1 MU/s, 3 MU/s, 5 MU/s, 8 MU/s and 10 MU/s) demonstrating that the introduced error of MUs per spot for a typical treatment would be less than 1%. The test Bragg Peak chamber was set to the iso-center with the voltage 400 V to collect the dose. The beam size enlarged and the beam density decreased, through the distance shifting from the vacuum window to the iso-center, when it is arrived at the connection plane. The collection efficiency of the Bragg Peak chamber with the 3 nA beam intensity at the iso-center was calculated which is better than 99.5%. The measured dosimetric results showed that the deviation of the normalized ratio of the Bragg Peak chamber readout and MU output is within ±1% at different dose rates. This is because the bias high voltage of the beamline monitor was set to −2500 V and the collection efficiency is almost not impacted by the ion recombination with high proton density. Table 4 shows the results of the proportionality test with an energy of 235 MeV using MU rates of 1 MU/s, 3 MU/s, 5 MU/s, 8 MU/s and 10 MU/s.

**Table 4:** The result of proportionality of MU delivery at 235 MeV.

Dose rate (MU/s)	TW34070 /IC output (nC/MU)	Maximum deviation (%)
1	4.3948	0.34
3	4.3975	0.29
5	4.3924	0.44
8	4.3971	0.46
10	4.4004	0.19

**Fig. 8:**
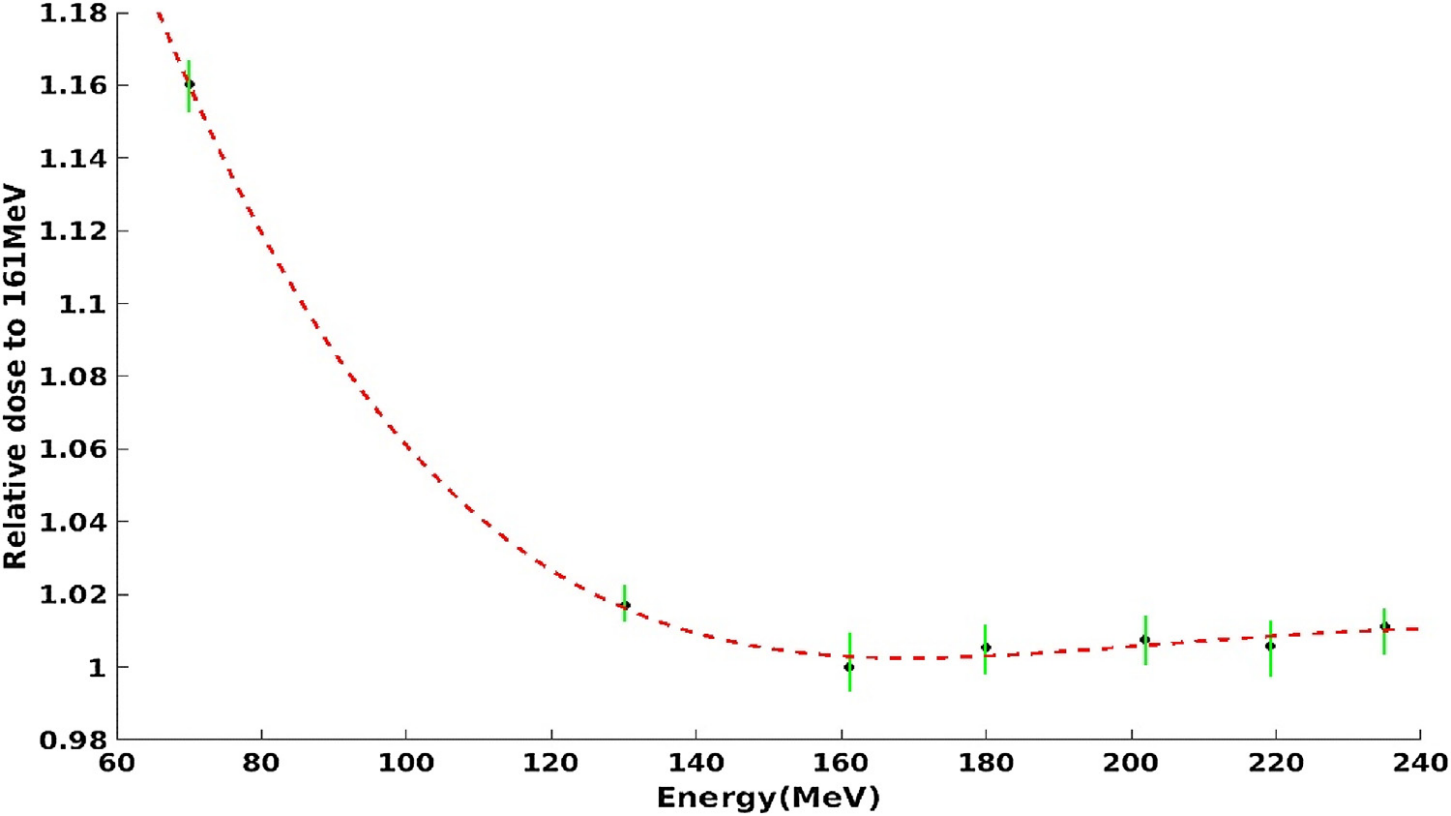
The monitor calibration factor normalized to 161.1 MeV.

Fig. 8 shows the values of the monitor calibration factor normalized to an energy of 161.1 MeV. The energies at 70 MeV, 130.1 MeV, 161.1 MeV, 179.9 MeV, 202 MeV, 219.2 MeV, and 235 MeV were measured and interpolated for the other energies that were not tested. The uniform scanning field size of 60 mm × 60 mm was delivered for the calibration of the monitor with the spacing set to 3 mm × 3 mm. The reference dosimeter used was a Markus chamber with a ^60^Co calibration factor in terms of absorbed dose to water provided by the NIM the primary standard dosimetry laboratories.

The absolute dose to water was calculated using the recommendations in TRS-398 as follows:

(1) D_meas_ (P_eff_)  = M_Q_ × N_D,W,Q0_ × k_Q,Q0_

Here, P_eff_ denotes the effective point of measurement in the proton beam and M_Q_ is the ion chamber reading corrected for influence quantities, N_D,W,Q0_ is the absorbed dose to water calibration coefficient of the ion chamber in a calibration beam of quality Q_0_ and k_Q,Q0_ is the beam quality correction factor to account for the use of the calibration coefficient in a different beam quality Q. By repeating the measurement at each energy ten times, it was found that the reproducibility of the results was better than 1%.

**Fig. 9:**
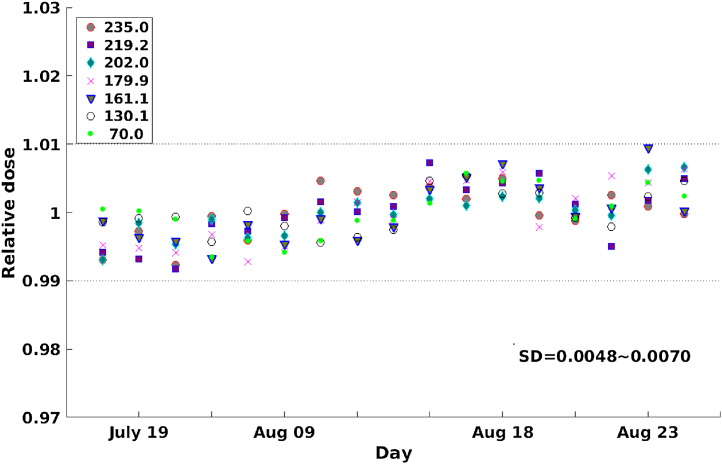
Monitor calibration factor measured daily result during the test period.

Fig. 9 shows a summary of measurements performed twice per day during the delivery system QA. These measurements were made once in the morning and once in the evening each day as part of the daily routine work. The result was very reproducible and stable exhibiting a one SD result of less than 1%.

## Conclusion

In this manuscript, the commissioning activities of the fixed beam line at SAPT were described including the calibration method for the primary monitor chamber with absolute dose to water. The performance tests and calibrations followed the IEC 62667 performance standard and the IAEA TRS-398 dosimetry recommendations. The results showed that the full width at half maximum for the beam spot size in air varied approximately from 6 mm to 13 mm. The depth dose in water with the measured DDF varied from 4.7 mm at 235 MeV to 0.7 mm at 70 MeV. This sharp DDF would be benefit to sparing the distal healthy organs in clinical application. The homogeneity of the scanned field was better than 2% for various energies as expected. Furthermore, the beam reproducibility and proportionality delivery accuracy was also stable with the results better than 0.1% and 1% respectively. Finally, the dose monitor calibration factor, its reproducibility, and stability were tested. Reproducibility tests exhibited a standard deviation (SD) result of less than 1% during the test period. All the measured dosimetric parameters showed that the design specifications were well achieved and the results are suitable for being used as a part of the clinical commissioning and quality assurance program for treating patients.
